# Supplementary material for: Effects of Glycated Glutenin Heat-Processing Conditions on Its Digestibility and Induced Inflammation Levels in Cells
Source: Foods. 2021 Jun 12;10(6):1365. doi: 10.3390/foods10061365 (PMC8231263; doi:10.3390/foods10061365)
Supplement: Supplementary file 1 [file foods-10-01365-s001.zip › foods-1220025-supplementary.pdf]

Supplementary Data

Effects of Glycated Glutenin Heat-processing Conditions on its Digestibility and Induced Inflammation Levels in Cells

Yaya Wang[1], Lu Dong[1], Yan Zhang[1], Junping Wang[2], Jin Wang[1], Wenwen Pang[1] and Shuo Wang[1*]

Table: 1

RAW264.7 cells (106 cells/well) were placed in a 6-well plate and cultured for 24 hours. After aspirating the medium, fresh medium supplemented with glutenin or glycated glutenin digestion products (10%, v/v) was added to the 6-well plate and cultured for 24 hours. After washing the wells with PBS, the total RNA was extracted with the TRIzo1 (Thermo Fisher Sientific, China) method. The microplate reader is used to determine the purity of the extracted RNA to ensure that the absorbance of the sample is between 1.8-2.1. LunaScriptTM SuperMix Kit (New England BioLabs, Massachusetts, USA) was used for cDNA synthesis. Finally, the genes that encode beta-actin and 18S were used as housekeeping genes. The primer sequences of β-actin, IL-6, TNF-α, IL-1β and RAGE were designed and shown in Supplementary Table S1.

Table S1. The primer sequences of β-actin, IL-6, TNF-α, IL-1β and RAGE

	Forward	Reverse
β-actin	ACAGCAGTTGGTTGGAGCAA	ACGCGACCATCCTCCTCTTA
IL-6	CTCTGGCGGAGCTATTGAGA	AAGTCTCCTGCGTGGAGAAA
TNF-α	CGTGGAACTGGCAGAAGAGG	CAGGAATGAGAAGAGGCTGAGAC
IL-1β	AAGGGCTGCTTCCAAACCTTTGAC	TGCCTGAAGCTCTTGTTGATGTGC
RAGE	AGTCCGAGTCTACCAGATTC	CATCTAAGTGCCAGCTAAGG
